# Use of Recombinant *Escherichia coli* Strains in Immunofluorescence Assays for Melioidosis Diagnosis

**DOI:** 10.3390/pathogens10050559

**Published:** 2021-05-06

**Authors:** Kanoknart Lantong, Jirarat Songsri, Sueptrakool Wisessombat, Wanida Mala, Warinda Prommachote, Wilaiwan Senghoi, Manas Kotepui, Jedsada Kaewrakmuk, Treenate Jiranantasak, Apichai Tuanyok, Wiyada Kwanhian Klangbud

**Affiliations:** 1 Biomedical Sciences Program, School of Allied Health Sciences, Walailak University, Nakhon Si Thammarat 80160, Thailand; kanoknath.namkl@gmail.com; 2 Center of Excellent Research for Melioidosis (CERM), School of Allied Health Sciences, Walailak University, Nakhon Si Thammarat 80160, Thailand; jirarat.so@wu.ac.th (J.S.); sueptrakool.wi@wu.ac.th (S.W.); wanida.ma@wu.ac.th (W.M.); warinda.pr@wu.ac.th (W.P.); wilaiwan.se@wu.ac.th (W.S.); 3 Medical Technology Program, School of Allied Health Sciences, Walailak University, Nakhon Si Thammarat 80160, Thailand; manas.ko@wu.ac.th; 4 Faculty of Medical Technology, Prince of Songkla University, Hat Yai, Songkhla 90110, Thailand; jedsada.k@psu.ac.th; 5 Department of Infectious Diseases and Immunology, College of Veterinary Medicine, University of Florida, Gainesville, FL 32611, USA; treenate@ufl.edu (T.J.); tuanyok@ufl.edu (A.T.)

**Keywords:** melioidosis, *Burkholderia pseudomallei*, *Escherichia coli*, immunofluorescence assay, whole-cell-based assay, TssM

## Abstract

*Burkholderia pseudomallei* is a Gram-negative bacterium and the causative agent of melioidosis in humans and animals in the tropics. The clinical manifestations of melioidosis are diverse, ranging from localized infections to whole-body sepsis. The effective serological method is crucial for the point-of-care diagnosis of melioidosis. The aim of this study was to develop indirect immunofluorescence assay (IFA)-based methods for detecting immunoglobulin G (IgG) antibodies in melioidosis patients. These methods use whole-cell antigens made from recombinant *E. coli* strains that express major *B. pseudomallei* antigens, including TssM, OmpH, AhpC, BimA, and Hcp1. A total of 271 serum samples from culture-confirmed melioidosis patients (*n* = 81), patients with other known infections (*n* = 70), and healthy donors (*n* = 120) were tested. Our study showed that the recombinant TssM strain had the highest performance, with 92.6% sensitivity, 100% specificity, 100% positive predictive value, 96.9% negative predictive value, 97.8% efficiency, 97.0% accuracy, and no cross-reactivity. The method agreement analysis based on *k* efficiency calculations showed that all five IFA methods perfectly agreed with the standard culturing method, while the traditional indirect hemagglutination (IHA) method moderately agreed with the culture. In summary, our investigations showed that the TssM-IFA method could be used for melioidosis diagnosis.

## 1. Introduction

*Burkholderia pseudomallei* is a Gram-negative bacterium, the etiological agent of melioidosis, a disease associated with high case-fatality rates in both animals and humans [1]. *B. pseudomallei* can be found in moist soil and fresh water in endemic areas, especially in Southeast Asia, South Asia, and Northern Australia. Melioidosis has been reported outside these endemic regions, such as in Brazil, New Caledonia in the Pacific Ocean, Mauritius in India, and elsewhere in America [2,3,4]. *B. pseudomallei* can infect animals and humans via skin inoculation, inhalation during severe weather events, and through contaminated water ingestion. Symptoms of melioidosis can be observed 2–4 weeks after exposure to the bacterium. However, the incubation period may last hours or years [5]. The clinical spectrum of melioidosis is broad and often mimics other illnesses, which can make diagnosis more challenging. Clinical presentations can range from skin and soft tissue abscesses to acute pneumonia and septicemia with fatal outcomes. In Northeast Thailand, the mortality rate is approximately 40%; however, it can reach as high as 90% in severe sepsis cases [6].

The culture method is a gold standard and reliable technique for melioidosis diagnosis. However, it is a time-consuming technique that usually takes at least 2–3 days for culturing and identification. Therefore, a rapid and convenient technique is needed for the point-of-care (POC) diagnosis of melioidosis. Efforts to develop and perform evaluations using POC diagnosis kits for the diagnosis of melioidosis continue to be published. For example, specific capsular polysaccharide detection kits for use with culture blood and other sample types such as the i-STAT cartridge [7] and Active Melioidosis Detect™ [8] have been developed, while for anti-Hcp1 (hemolysin co-regulated protein 1) antibody detection, recent developments include the Melioidosis DS [9] and Hcp1-ICT [10]. Hcp1 is a structural component and substrate of a type 6 secretion system and can be expressed in vivo. A high level of anti-Hcp1 IgG can discriminate melioidosis cases from healthy donors. Moreover, anti-Hcp1 is highest in the acute phase and declines after week 52 post recovery [11]. Phokrai and colleague demonstrated performances of Hcp-1 ICT for serological melioidosis diagnosis with 83.3% sensitivity and 86.1 to 100% specificity [11]. According to a literature review, antibody detection is still necessary for melioidosis diagnosis because it takes less time than a culture. The currently used serological methods include the indirect hemagglutination assay (IHA), immunofluorescence assay (IFA), enzyme-linked immunosorbent assay (ELISA), and lateral flow immunochromatography assay (LFI) [12]. These methods use crude antigens, purified antigens, or whole *B. pseudomallei* cells. However, the results vary and they depend on the antigen selection for each assay [13,14]. For example, in a study by Harris and colleagues, the IHA method used crude antigens that were prepared from 20 strains of *B. pseudomallei* to sensitize sheep red-blood cells [15], and they also demonstrated that the performance was not satisfied, with 51% sensitivity and 93% specificity [16]. In addition, an IFA method that used whole *B. pseudomallei* cells as antigens also had a low specificity and sensitivity [17]. Our current study developed an alternative IFA method that used recombinant *E. coli* strains that expressed major *B. pseudomallei* antigens as the whole-cell antigens. This was for safe handling and could be shared among laboratories without restriction. These major protein antigens included alkyl hydroperoxide reductase (AhpC), Burkholderia intracellular motility factor A (BimA), outer-membrane protein H (OmpH), hemolysin co-regulated protein 1 (Hcp1), and Type VI secretion-system protein M (TssM). AhpC is relatively strong, with a constant antibody response over a prolonged period over 52 weeks after infection [18]. Burkholderia intracellular motility factor A (BimA) is associated with host actin dynamics that enable bacterial movement between eukaryotic cells and the evasion of immune surveillance [19]. Outer-membrane protein H (OmpH) is also a potential antigen for serological diagnosis due to its high immunogenicity, which has been reported in previous studies [20,21]. Hemolysin co-regulated protein I (Hcp1) is a major virulence factor that plays a critical role in the intracellular lifestyle of *B. pseudomallei,* and is also known to be a T-cell-dependent protein antigen [14]. TssM, a deubiquitinase of the Type VI secretion system (TssM) known to inhibit NF-kB and IFN-β, is expressed and likely secreted in humans [22,23], thus it can stimulate antibody production in the early phase of infection well. The main reason we selected recombinant *E. coli* strains for IFA development is that they are safe for handling and sharing among laboratories. This will enable them to be used in the diagnosis of melioidosis in the community, as recombinant *E. coli* are easy to propagate in-house compared with other assay kits.

The use of whole-cell bacteria-based kits for clinical and environmental detections has been reported in several studies. For example, Kylilis and colleagues demonstrated that *E. coli* whole-cell antifibrinogen fragment expressions could be used as particles for blood fibrinogen detection with an agglutination assay [24]. Due to its low cost and high effectiveness, the whole-cell-based approach provides a greater opportunity to be developed and applied in many analyses. Thus, we aimed to validate the performance methods of these IFAs for the detection of antibody titers in melioidosis patients.

## 2. Results

### 2.1. Effect of Serum Preabsorbed Step

**Figure 1:**
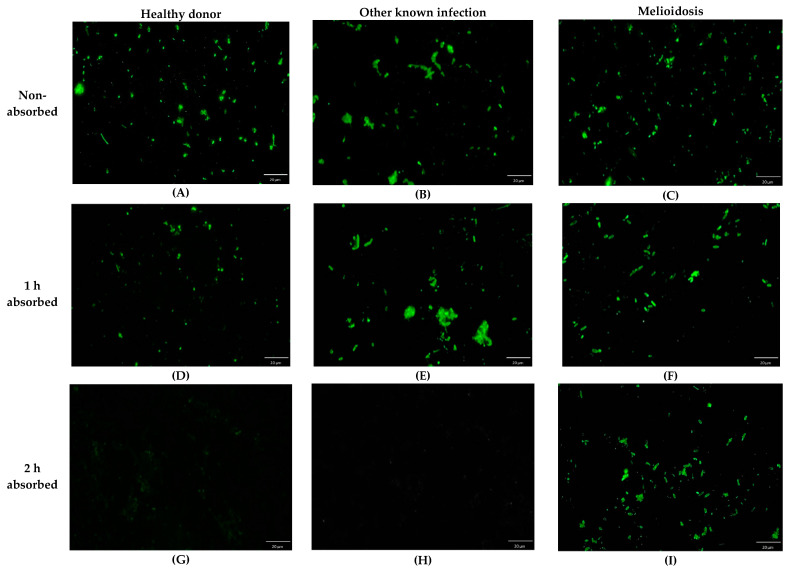
Whole cell-based indirect immunofluorescence assay (IFA) slides visualized under an immunofluorescence microscope for anti-*B. pseudomallei* antibody detection when tested and diluted 1:16 for a healthy donor (**A**,**D**,**G**), another known infection (*Pseudomonas* spp.) (**B**,**E**,**H**), and melioidosis (**C**,**F**,**I**) with *E. coli*-TssM indicating nonabsorbed, 1 h absorbed, and 2 h absorbed serum, respectively.

The nonabsorbed serum of the culture-confirmed melioidosis cases, other known infections, and healthy donors’ sera expressed nonspecific antigen antibodies that reacted to *E. coli* surface antigens. The fluorescence grew when we tested nonabsorbed serum with *E. coli* strains expressing *B. pseudomallei* antigens TssM, OmpH, AhpC, BimA, or Hcp1. After 2 h of absorption, the nonspecific reaction problem was eliminated in healthy donors and those with other known infections, but was still shown to be positive in culture-confirmed melioidosis cases. Examples of the IFA visualization under a fluorescence microscope are shown in Figure 1.

### 2.2. Cutoff Value of Indirect Immunofluorescence Assay

**Figure 2:**
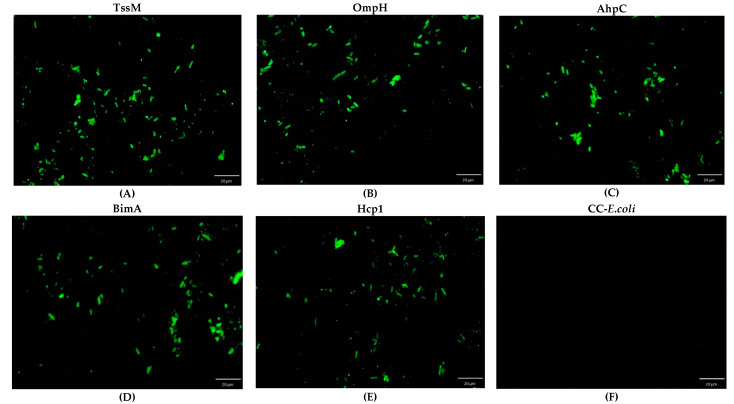
Whole cell-based IFA slides for anti-*B. pseudomallei* antibody detection at a titer 1:16 for a melioidosis patient visualized under an immunofluorescence microscope: *E. coli*-TssM gene (**A**), *E. coli*-OmpH (**B**), *E. coli*-AhpC (**C**), *E. coli*-BimA (**D**), *E. coli*-Hcp1 (**E**), and CC-*E. coli* (cell control) (**F**).

**Figure 3:**
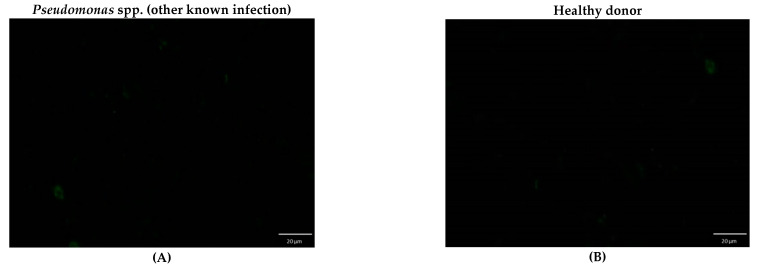
Whole cell-based IFA slides for anti-*B. pseudomallei* antibody detection at a titer of 1:16 for another known infection patient (**A**) and a healthy donor (**B**) visualized under an immunofluorescence microscope.

All five recombinant *E. coli* strains expressing *B. pseudomallei* antigens TssM, OmpH, AhpC, BimA, or Hcp1 were used with IFA to detect IgG titers in culture-confirmed melioidosis cases, healthy donors, and other nonmelioidosis patients. Examples of IFA visualization under a fluorescence microscope are shown in Figure 2 and Figure 3. The highest area under curve (AUC) values for most of the antigens were at titer 1:16. The ROC curve (receiver operating characteristic curve) of each antigen was shown in supplementary Figures S1–S5. This suggests that a titer of 1:16 could be used as a cutoff titer for positive results for all of the IFAs shown in Table 1.

### 2.3. Method Performances

Using a cutoff titer of 1:16, as described above, 75 out of 81 (92.6%) of the serum samples from culture-confirmed melioidosis patients were positive with TssM-IFA, while 72 (88.9%), 69 (85.2%), 64 (79%), and 60 (61.7%) samples were positive with the OmpH-, AhpC-, BimA-, and Hcp1-IFAs, respectively. When higher cutoff titer values were used, the percentage of the positive results was reduced (Table 1). We noted that TssM-IFA had the highest positivity, of up to 46.9%, when the 1:64 titer was used.

All 120 sera from healthy individuals and 70 sera from nonmelioidosis patients showed negative results at a cutoff titer of 1:16 (Table 2).

Using a cutoff titer of 1:160 for IHA, as recommended by the test kit and a recent study [25], 30 (37.0%) serum samples from the culture-confirmed melioidosis patients were positive with this technique, while all 120 samples from the healthy donors were negative. One sample from a patient who was known to be infected with *Acinetobacter* spp. showed a false-positive with IHA with a titer of 1:320 (1.43%). The data are shown in Table 3.

The method performances of IFA and IHA were compared based on sensitivity, specificity, positive predictive value (PPV), negative predictive value (NPV), rate of cross-reactivity, efficiency, and accuracy. 

With a 1:16 cutoff titer for the IFA method, all five assays had different calculated performances, as shown in Table 4. We noted that TssM-IFA had the highest performance with a 92.6% sensitivity, 100% specificity, 100% PPV, 96.9% NPV, 97.8% efficiency, and 97.0% accuracy, while Hcp1-IFA had the poorest performance, with a 61.72% sensitivity, 100% specificity, 100% PPV, 85.97% NPV, 88.56% efficiency, and 88.0% accuracy. All five assays had 100% specificity with various sensitivities. They were no cross-reactions for any of the five IFAs with serum samples from other known infected patients and healthy donors. On the other hand, when a 1:160 cutoff titer was used, IHA had lower method performances, including 37.0% sensitivity, 99.5% specificity, 69.7% PPV, 78.8% NPV, 80.8% efficiency, 80.0% accuracy, and 1.4% cross-reactivity. There were statistically significant differences in sensitivity between IHA compared to TssM-, OmpH-, AhpC-, BimA-, and Hcp1-IFA (*p* < 0.05). Moreover, the sensitivity of TssM-IFA showed nonsignificant differences only with OmpH, but significant differences for AhpC-, BimA-, and Hcp1-IFA (*p* = 0.041, 0.003, and 0.000, respectively).

### 2.4. The Method Agreement

The kappa coefficient (*k*) was used to determine the level of method agreement between each assay and the culture. The *k* values were calculated as 0.95, 0.91, 0.88, 0.84, and 0.80 for TssM-, OmpH-, AhpC-, BimA-, and Hcp1–IFA, respectively (Table 4). These values were in the range of 0.81–1.00, the perfect range of agreement between these five IFAs with a culture conformation of melioidosis. The *k* value of IHA was determined to be 0.41, which is in the range of 0.41–0.60, the moderate range of agreement for the culture. 

## 3. Discussion

The current serological diagnostic tests for identifying *B. pseudomallei* infection cannot provide satisfactory sensitivity and specificity. Although culture remains a standard laboratory diagnosis for melioidosis, it still has some disadvantages as it is time-consuming. The severity and high mortality rate of melioidosis are of major concern in countries where melioidosis is endemic. Thus, there is a critical need for easy, fast, highly sensitive, highly specific, and accurate melioidosis diagnosis [26]. Previous studies showed the many advantages of using recombinant proteins over whole crude antigens for the serological diagnosis of melioidosis [27,28,29]. Therefore, this study aimed to develop a reliable method for detecting an antibody titer against *B. pseudomallei* antigens in patients’ sera. As using live *B. pseudomallei* strains to make whole-cell antigens for serological diagnosis test is too dangerous for laboratorians, we decided to use the whole cell of the recombinant *E. coli* strains that expressed the major *B. pseudomallei* antigens instead.

In this study, candidate recombinant *B. pseudomallei* proteins that expressed *E. coli* were used as antigens of the indirect immunofluorescence method for melioidosis antibody detection. The conjugated goat antihuman IgG-FITC was a secondary antibody used for detection. Therefore, only specific anti-*B. pseudomallei* IgG antibodies were detected. This could be a limitation of this study. However, anti-*B. pseudomallei* IgM detection by the same IFA method was very interesting to study further. It might be helpful for detection in the acute phase of melioidosis infection. The earlier detection of infectious disease might reduce the mortality rate [30].

The best performance antigen is TssM, which expresses the highest sensitivity, negative predictive value (NPV), efficiency, and accuracy. TssM, a deubiquitinase of the Type VI secretion system (TssM) known to inhibit NF-*k*B and IFN-β, is expressed and likely secreted during human infection [22]; thus, it can stimulate antibody production in the early phase of infection well. Tan and colleagues confirmed that 60% of acute or subacute melioidosis patients presented antibodies against TssM in the sera within 2 weeks after hospital admission [22]. Hemolysin-coregulated protein (Hcp1) is a serological candidate target that was previously developed for lateral-flow rapid diagnosis [9,11] and ELISA [31]; however, our findings demonstrate that the Hcp1 performances (sensitivity, NPV, and accuracy) and method agreement (*k*) are lower than TssM. No previous report has ever directly compared the potential of TssM and Hcp1 for melioidosis diagnosis in the same approach. Thus, our study is the first to mention that TssM has a better performance than Hcp1 for serological diagnosis by whole-cell-based IFA assay. According to the high performance of Hcp1 in previous studies and TssM in a recent study, the performance of mixing both antigens through whole-cell-based IFA assay is interesting to further study.

Our study is also the first to use recombinant whole-cell-based *B. pseudomallei* proteins expressing *E. coli* as antigens for melioidosis diagnosis with the IFA method. They are many advantages to using this approach for melioidosis detection, including being easy and safe to manipulate. We cultured *E. coli* in a BLS 2 laboratory, while BSL2-enhanced or BSL3 laboratories should be used for *B. pseudomallei* study [26]. The kappa coefficient (*k*) of this approach was in perfect agreement with the standard culture method and the moderate agreement with the IHA with culture. This indicated that this whole-cell-based IFA method’s performance was better than that of the IHA method.

## 4. Materials and Methods

### 4.1. Bacterial Strains, Plasmids, and Culture Conditions

Five recombinant *E. coli* BL21 (DE3) strains, which were expressed as AhpC (BPSS2096), BimA (BPSS1492), OmpH (BPSL2150), Hcp1 (BPSS1498), and TssM (BPSS1512), were made. The cloning protocols followed, but modified, a previous study [32]. Briefly, the DNA sequences of *ahpC, bimA, ompH, hcp1,* and *tssM* were amplified from the genomic DNA of *B. pseudomallei* strain K96243 using primers that introduced *Sal*I and *Nde*I sites. The PCR product was cut by restriction enzymes (REs), then ligated to pET-21b, which cut the same REs. The primers were designed to remove the secretion signal sequences. Inserts were verified through digest and Sanger sequencing. Each recombinant plasmid was then transformed into *E. coli* BL21 (DE3). Each recombinant strain was grown at 37 °C on Luria–Bertani (LB) agar or in LB broth (Merck, Darmstadt, Germany) supplemented with 100 µg/mL of ampicillin, overnight.

### 4.2. Human Serum Samples and Preparation

Leftover sera from routine patient diagnoses were collected from four provincial hospitals in Thailand. These included 81 serum samples from culture-confirmed melioidosis patients diagnosed at Buriram hospital (*n* = 40), Mukdahan hospital (*n* = 15), Chaiyaphum hospital (*n* = 19), and Trang hospital (*n* = 7). In addition, 70 samples were from patients who were diagnosed at Thasala hospital in Nakhon Si Thammarat province, with known infections caused by various other bacterial and viral pathogens, such as *Pseudomonas* spp. (*n* = 4), *Staphylococcus* spp. (*n* = 10), *Streptococcus* spp. (*n* = 20), *Escherichia coli* (*n* = 8), Bacillus spp. (*n* = 6), *Klebsiella* spp. (*n* = 2), *Acinetobacter* spp. (*n* = 2). *Proteus* spp. (*n* = 2), *Salmonella* spp. (*n* = 2), Hepatitis B virus (HBV; *n* = 3), and Human Immunodeficiency virus (*n* = 11). Moreover, 120 serum samples from healthy donors were collected from the Blood Bank at Maharat-Nakhon Si Thammarat hospital, Nakhon Si Thammarat province. The serum samples were stored at −20 ℃ until their required use.

Bacterial suspension made from an *E. coli* BL21 (DE3) culture was used to absorb nonspecific antibodies. To make the bacterial suspension, a single colony of *E. coli* grown on a nutrient agar plate was inoculated into a tube containing 5 mL of LB broth, and the tube was incubated at 37 ℃ for 24 h. A volume of 5 mL of bacterial culture was centrifuged at 300× *g* for 5 min. The cell pellet was collected and the supernatant was discarded. The cell pellet had 5 mL of 4% paraformaldehyde added, and the tube was rotated at room temperature for 1 h prior to centrifugation at 300× *g* for 5 min. The supernatant was discarded, and the tube had approximately 2 mL of sterile normal saline added to obtain a 0.5 McFarland standard concentration. To absorb the nonspecific antibodies in the serum, 100 µL of serum was mixed well with equal volume paraformaldehyde fixed *E.coli* in a 1.5 mL microtube, and incubated at room temperature for 2 h. The sample was centrifuged at 6000× *g* for 15 min, and the supernatant was analyzed by IFAs with a magnification of 100×.

### 4.3. Preparation of the Whole-Cell Antigens from the Recombinant E. coli for IFA

Each recombinant *E. coli* strain was subcultured onto a nutrient agar plate (Merck, Darmstedt, Germany), and incubated at 37 °C overnight. The next day, a single colony of the recombinant *E. coli* strain was inoculated into a tube containing 4 mL of trypticase broth (Merck, Darmstedt, Germany) supplemented with 0.1 mM Isopropyl β-d-1-thiogalactopyranoside (IPTG) (Merck, Darmstedt, Germany), and was incubated at 37 °C, overnight. The tube was centrifuged at 6000× *g* for 15 min, and the cell pellet was collected. A volume of 5 mL of 4% paraformaldehyde was added and incubated at 4 °C for 48 h to fix the bacterial cells. The tube was centrifuged at 6000× *g* for 15 min and the supernatant was discarded. The pellet was washed once with 1 mL of 1 × PBS, pH 7.3, and the turbidity was adjusted to the 0.5 McFarland standard. The paraformaldehyde-fixed cells were stored at 4 °C until their use in IFA assays.

### 4.4. Indirect Immunofluorescence Assay (IFA)

The IFA assays followed the methods described by Wuthiekanun and colleagues [33], and Vadivelu and colleagues [17], with some modifications. Briefly, a Teflon™ multiple-well microscope slide (Thermo Fisher Scientific, Waltham, MA, USA) was cleaned by rinsing with 75% ethanol. Each test well was filled with 20 µL of the prepared antigens. The slide was air-dried and fixed with cold acetone (1:1 v/v). The coated slide was dried in a laminar flow hood and then kept at −20 °C until use. Two-fold serial dilutions of the serum sample were made using PBS until a dilution of 1:64 was obtained. A volume of 7 µL for each diluted serum sample was added to a well of the coated slide. The slide was then incubated in a moist chamber at 37 °C for 30 min, washed three times with a PBS buffer (pH 7.4), for 10 min, and left to dry at room temperature. A volume of 7 µL of 1:50 (v/v) FITC-conjugated anti-human IgG (Thermo Fisher Scientific, Waltham, MA, USA) in PBS was added to the reaction. The slide was incubated for 30 min and washed three times with PBS at pH 7.4. The slide was air-dried and mounted with glycerol and covered with a coverslip prior, for a visualization under a fluorescence microscope with a magnification of 100×. The IFA titer of each melioidosis patients’, other known infection and healthy donor samples when use TssM, OmpH, AhpC, BimA, and Hcp1 expressed *E. coli* as an antigen was shown in supplementary Tables S1–S3, respectively.

### 4.5. Indirect Hemagglutination Assay (IHA)

The IHA technique was described previously by Vadivelu and colleagues [34]. In this study, the IHA kit was purchased from the National Institute of Health of Thailand (Ministry of public health, Thailand). According to the vendor’s protocol, the serum sample was inactivated at 56 °C for 30 min, and heterophile antibodies were absorbed with 5% unsensitized sheep red-blood cells (SRBCs) at room temperature for 1 h. The absorbed serum was 2-fold serially diluted in a 96-well plate. The dilutions were made from 1:10 to 1:5120 concentration in 0.15 M PBS containing 0.5% bovine serum albumin. Each well contained 50 µL of absorbed serum. A volume of 25 µL of 1% sensitized sheep red-blood cells was added to each well except for the control well, to which the unsensitized SRBCs was added. The plate was covered and sealed with aluminum foil tape, gently shaken for 2 min and incubated for 2 h at room temperature. The resulting agglutination was observed with naked eyes. The titer of the last dilution that showed more than 50% of the hemagglutination was recorded.

### 4.6. Statistical Analyses

Statistical analyses were performed using diagnostic tests, and the sensitivity, specificity, positive predictive value (PPV), negative predictive value (NPV), efficiency, accuracy, cross-reactivity rate, and kappa coefficient (*k*) of the IFA and IHA assays were compared [35]. The 2 × 2 contingency table that was used to calculate the performance methods is shown in Table 5. The examination of the melioidosis and nonmelioidosis patients was performed by quantifying the relative operative cutoff (ROC) and area under curves (AUCs) analyses of different serum titers and different antigens [36]. The data were statistically analyzed using IBM^®^ SPSS^®^ statistics version 23. The method performances and method agreement (kappa coefficient; *k*) were calculated using Equations (1)–(8), as shown below. The strange of agreement was interpreted by *k* value according to Table 6. McNemar’s test was used for method performance comparisons with a 95% confidential interval.

% Sensitivity = {a / (a + c)} × 100(1)

% Specificity = {c / (d + c)} × 100(2)

% Positive predictive value (PPV) = {a / (a + b)} × 100(3)

% Negative predictive value (NPV) = {d / (d + c)} × 100(4)

% Efficiency = {(a + d) / *n*} × 100 (5)

% Accuracy = {a + d / (a + b + c + d)} × 100 (6)

% Cross-reactivity = (positive samples / samples may react with the reagent) × 100(7)

Kappa coefficient (*k*) = 2 {(a × d) – (b × c)} / {(a + b) (b + d) + (a + c) (c + d)} (8)

## 5. Conclusions

Recombinant *B. pseudomallei* proteins expressing *E. coli*, namely TssM, OmpH, AhpC, BimA, and Hcp1, are used as whole-cell-based antigens for the indirect immunofluorescence (IFA) method for melioidosis antibody detection. The appropriate cutoff value for the developed IFA method is 1:16. The best performance antigen is TssM, with 92.59% sensitivity, 100% specificity, 100% PPV, 96.93% NPV, 97.78% efficiency, and 97.0% accuracy. The performance antigens sorted according to their method performances were OmpH, AhpC, BimA, and Hcp1, respectively. The method reliability of TssM whole-cell-based antigen-IFA is perfectly in agreement with the reference culture method (*k* = 0.95). In addition, the remaining 4 candidate antigens also have suitable *k* values in the range of 0.81–1.00.

## Figures and Tables

**Table 1:** The area under curve (AUC) calculated from the relative operative cutoff (ROC) curve of different titer with different whole-cell-based IFA antigens.

Titer	Area Under Curve (AUC)				
	TssM	OmpH	AhpC	BimA	Hcp1
≤1:8	0.958	0.918	0.908	0.895	0.804
1:16	0.963	0.942	0.929	0.910	0.811
1:32	0.963	0.942	0.929	0.910	0.811
≥1:64	0.963	0.942	0.929	0.910	0.811

**Table 2:** Anti-*B. pseudomallei* antibody detection from different titers for all three sets of serum sample groups with different whole-cell-based IFA antigens.

Serum Samples	Total No.	Number of Sample Positive with Antibody Titer																			
		TssM				OmpH				AhpC				BimA				Hcp1			
		1:8	1:16	1:32	1:64	1:8	1:16	1:32	1:64	1:8	1:16	1:32	1:64	1:8	1:16	1:32	1:64	1:8	1:16	1:32	1:64
Culture-confirmed melioidosis patients	81	75	75	65	38	72	72	42	17	70	69	38	7	67	64	28	6	51	50	20	3
Other known infections:	70	2	0	0	0	10	0	0	0	9	0	0	0	7	0	0	0	4	0	0	0
*Pseudomonas* spp.	4	2	0	0	0	0	0	0	0	1	0	0	0	0	0	0	0	1	0	0	0
*Staphylococcus* spp.	10	1	0	0	0	2	0	0	0	2	0	0	0	0	0	0	0	0	0	0	0
*Streptococcus* spp.	20	1	0	0	0	2	0	0	0	2	0	0	0	2	0	0	0	2	0	0	0
*Escherichia coli*	8	0	0	0	0	2	0	0	0	1	0	0	0	1	0	0	0	1	0	0	0
*Bacillus* spp.	6	0	0	0	0	1	0	0	0	0	0	0	0	1	0	0	0	0	0	0	0
*Klebsiella* spp.	2	0	0	0	0	0	0	0	0	1	0	0	0	0	0	0	0	0	0	0	0
*Acinetobacter* spp.	2	0	0	0	0	1	0	0	0	0	0	0	0	0	0	0	0	0	0	0	0
*Proteus* spp.	2	0	0	0	0	0	0	0	0	1	0	0	0	0	0	0	0	0	0	0	0
*Salmonella* spp.	2	0	0	0	0	0	0	0	0	0	0	0	0	1	0	0	0	0	0	0	0
Hepatitis B virus (HBV)	3	0	0	0	0	0	0	0	0	1	0	0	0	0	0	0	0	0	0	0	0
Human Immunodeficiency virus (HIV)	11	0	0	0	0	2	0	0	0	0	0	0	0	2	0	0	0	0	0	0	0
Healthy donors	120	0	0	0	0	0	0	0	0	0	0	0	0	0	0	0	0	0	0	0	0

**Table 3:** Anti-*B. pseudomallei* antibody detection by indirect hemagglutination assay (IHA) for different serum sources.

Serum Samples	Total No.	Antibody Titer by IHA Assay									
		≤1:10	1:20	1:40	1:80	1:160	1:320	1:640	1:1280	1:2560	≥1:5120
Culture-confirmed melioidosis patients	81	39	4	4	3	8	11	5	3	1	2
Other known infections:	70	67	0	1	1	0	1	0	0	0	0
*Pseudomonas* spp.	4	4	0	0	0	0	0	0	0	0	0
*Staphylococcus* spp.	10	9	0	1	0	0	0	0	0	0	0
*Streptococcus* spp.	20	20	0	0	0	0	0	0	0	0	0
*Escherichia* coli	8	8	0	0	0	0	0	0	0	0	0
*Bacillus* spp.	6	6	0	0	0	0	0	0	0	0	0
*Klebsiella* spp.	2	2	0	0	0	0	0	0	0	0	0
*Acinetobacter* spp.	2	1	0	0	0	0	0	0	0	0	0
*Proteus* spp.	2	1	0	0	0	0	1	0	0	0	0
*Salmonella* spp.	2	2	0	0	1	0	0	0	0	0	0
Hepatitis B virus (HBV)	3	3	0	0	0	0	0	0	0	0	0
Human Immunodeficiency virus (HIV)	11	11	0	0	0	0	0	0	0	0	0
Healthy donors	120	117	2	0	1	0	0	0	0	0	0

**Table 4:** Method performances and method agreement (*k*) of whole-cell-based IFA and IHA assays when the culture method is the reference method.

Performances	IFA (Cut-Off ≥ 1:16)					IHA (Cut-Off ≥ 1:160)
	TssM	OmpH	AhpC	BimA	Hcp1	
Sensitivity (%)	92.6	88.9	85.2	79.0	61.7	37.0
Specificity (%)	100.0	100.0	100.0	100.0	100.0	99.5
PPV (%)	100.0	100.0	100.0	100.0	100.0	69.77
NPV (%)	96.9	95.0	94.1	91.9	86.0	78.8
Efficiency (%)	97.8	96.7	95.5	93.7	88.6	80.8
Accuracy (%)	97.0	96.0	95.0	93.0	88.0	80.0
Cross-reactivity (%)	0	0	0	0	0	1.4
Kappa coefficient (*k*)	0.95	0.91	0.88	0.84	0.80	0.41

**Table 5:** A 2 × 2 contingency table illustrating the outcomes of a comparison between an alternative method (IFA or IHA) and the gold-standard culture method.

		Standard Culture Method	
		Positive	Negative
**Alternative Method** **(IFA or IHA)**	**Positive**	a (True positive)	b (False positive)
	**Negative**	c (False negative)	d (True negative)

**Table 6:** The strange of agreement is interpreted considering the kappa coefficient.

Kappa Value (*k*)	Strange of Agreement
<0.00	Poor
0.00–0.20	Slight
0.21–0.40	Fair
0.41–0.60	Moderate
0.61–0.80	Substantial
0.81–1.00	Almost Perfect

## Supplementary Materials

The following are available online at https://www.mdpi.com/article/10.3390/pathogens10050559/s1. Table S1: Result Anti-*B. pseudomallei* antibody detection from sera of melioidosis patients’ samples when testing IFA that use TssM, OmpH, AhpC, BimA, and Hcp1 expressed *E. coli* as an antigen. Table S2: Results of anti-*B. pseudomallei* antibody detection from sera of other known infection samples when testing IFA that use TssM, OmpH, AhpC, BimA, and Hcp1 expressed *E.coli* as an antigen. Table S3: Results of anti-*B. pseudomallei* antibody detection from the sera of healthy donor samples when testing IFA that use TssM, OmpH, AhpC, BimA, and Hcp1 expressed *E.coli* as an antigen. Figure S1: ROC-AUC analysis of TssM-IFA. Figure S2: ROC-AUC analysis of OmpH-IFA. Figure S3: ROC-AUC analysis of AhpC-IFA. Figure S4: ROC-AUC analysis of BimA-IFA. Figure S5: ROC-AUC analysis of Hcp1-IFA.
