# Supplementary material for: Use of Recombinant Escherichia coli Strains in Immunofluorescence Assays for Melioidosis Diagnosis
Source: Pathogens. 2021 May 6;10(5):559. doi: 10.3390/pathogens10050559 (PMC8148196; doi:10.3390/pathogens10050559)
Supplement: Supplementary file 1 [file pathogens-10-00559-s001.zip › pathogens-1180248-supplementary figures.pdf]

TssM-IFA

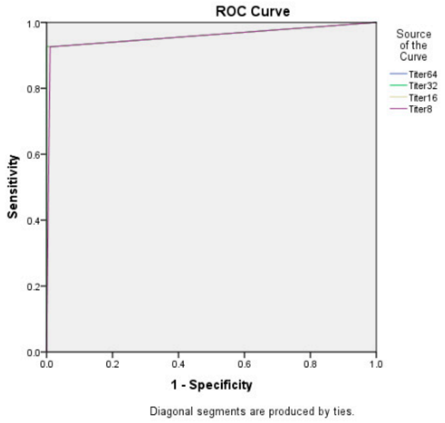

Case Processing Summary

Diagnosis	Valid N (listwise)
Positive[a]	81
Negative	190

Larger values of the test result variable(s) indicate stronger evidence for a positive actual state.

a. The positive actual state is Disease.

Area Under the Curve

Test Result Variable(s)	Area
Titer64	.963
Titer32	.963
Titer16	.963
Titer8	.958

The test result variable(s): Titer64, Titer32, Titer16, Titer8 has at least one tie between the positive actual state group and the negative actual state group. Statistics may be biased.

Figure S1. ROC-AUC analysis of TssM-IFA.

OmpH-IFA

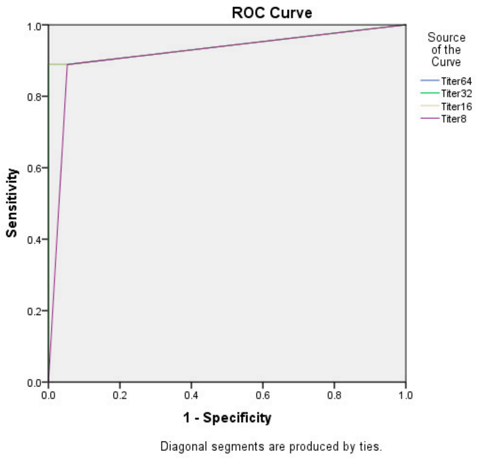

Case Processing Summary

Diagnosis	Valid N (listwise)
Positive[a]	81
Negative	190

Larger values of the test result variable(s) indicate stronger evidence for a positive actual state.

a. The positive actual state is Melioid.

Area Under the Curve

Test Result Variable(s)	Area
Titer64	.942
Titer32	.942
Titer16	.942
Titer8	.918

The test result variable(s): Titer64, Titer32, Titer16, Titer8 has at least one tie between the positive actual state group and the negative actual state group. Statistics may be biased.

Figure S2. ROC-AUC analysis of OmpH-IFA.

AhpC-IFA

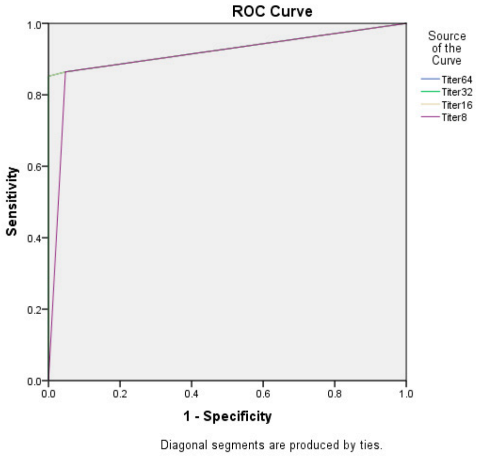

Case Processing Summary

Diagnosis	Valid N (listwise)
Positive[a]	81
Negative	190

Larger values of the test result variable(s) indicate stronger evidence for a positive actual state.

a. The positive actual state is Melioid.

Area Under the Curve

Test Result Variable(s)	Area
Titer64	.929
Titer32	.929
Titer16	.929
Titer8	.908

The test result variable(s): Titer64, Titer32, Titer16, Titer8 has at least one tie between the positive actual state group and the negative actual state group. Statistics may be biased.

Figure S3. ROC-AUC analysis of AhpC-IFA.

BimA-IFA

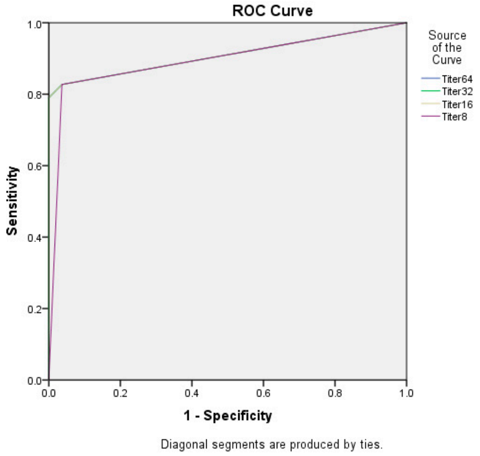

Case Processing Summary

Diagnosis	Valid N (listwise)
Positive[a]	81
Negative	190

Larger values of the test result variable(s) indicate stronger evidence for a positive actual state.

a. The positive actual state is Melioid.

Area Under the Curve

Test Result Variable(s)	Area
Titer64	.910
Titer32	.910
Titer16	.910
Titer8	.895

The test result variable(s): Titer64, Titer32, Titer16, Titer8 has at least one tie between the positive actual state group and the negative actual state group. Statistics may be biased.

Figure S4. ROC-AUC analysis of BimA-IFA.

HcpI-IFA

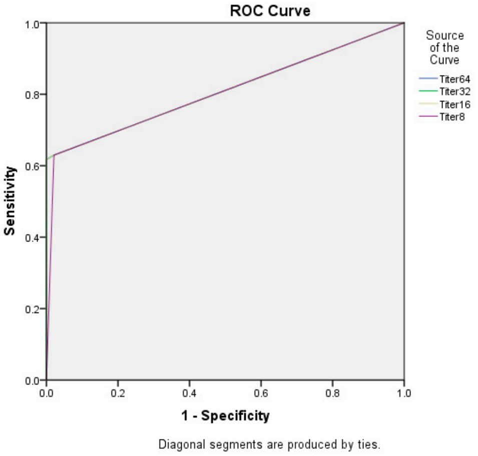

Case Processing Summary

Diagnosis	Valid N (listwise)
Positive[a]	81
Negative	190

Larger values of the test result variable(s) indicate stronger evidence for a positive actual state.

a. The positive actual state is Melioid.

Area Under the Curve

Test Result Variable(s)	Area
Titer64	.811
Titer32	.811
Titer16	.811
Titer8	.804

The test result variable(s): Titer64, Titer32, Titer16, Titer8 has at least one tie between the positive actual state group and the negative actual state group. Statistics may be biased.

Figure S5. ROC-AUC analysis of HcpI-IFA.
