# Supplementary material for: Use of Recombinant Escherichia coli Strains in Immunofluorescence Assays for Melioidosis Diagnosis
Source: Pathogens. 2021 May 6;10(5):559. doi: 10.3390/pathogens10050559 (PMC8148196; doi:10.3390/pathogens10050559)
Supplement: Supplementary file 1 [file pathogens-10-00559-s001.zip › pathogens-1180248-supplementary tables.pdf]

Table S1. Result Anti-*B. pseudomallei* antibody detection from serums of Melioidosis patients samples when testing by IFA that use TssM, OmpH, AhpC, BimA, and Hcp1 expressed *E. coli* as antigen

**Number**	**IFA-Titers**																			
	TssM				OmpH				AhpC				BimA				Hcp1			
	1:8	1:16	1:32	1:64	1:8	1:16	1:32	1:64	1:8	1:16	1:32	1:64	1:8	1:16	1:32	1:64	1:8	1:16	1:32	1:64
**Buriram hospital**																				
Bu1	+	+	+	+	+	+	+	+	+	+	+	0	+	+	+	0	+	+	0	0
Bu2	+	+	0	0	+	+	0	0	+	+	0	0	+	+	0	0	0	0	0	0
Bu3	+	+	+	0	+	+	+	+	+	+	+	0	+	+	0	0	+	+	0	0
Bu4	+	+	+	+	+	+	+	+	+	+	+	+	+	+	+		+	+	+	0
Bu5	+	+	+	0	+	+	+	+	+	+	+	0	+	+	0	0	+	+	0	0
Bu6	+	+	+	0	+	+	0	0	+	+	0	0	+	+	0	0	+	+	0	0
Bu7	+	+	+	+	+	+	+	+	+	+	+	0	+	+	+	+	+	+	+	+
Bu8	+	+	+	0	+	+	0	0	+	+	0	0	+	+	0	0	+	+	0	0
Bu9	+	+	+	+	+	+	+	+	+	+	+		+	+	0	0	+	+	+	0
Bu10	0	0	0	0	0	0	0	0	0	0	0	0	0	0	0	0	0	0	0	0
Bu11	+	+	+	0	+	+	0		+	+	+	0	+	+	+	0	+	+	+	0
Bu12	+	+	+	+	+	+	+	+	+	+	+	0	+	+	+	0	+	+	0	0
Bu13	+	+	+	+	+	+	+	+	+	+	+	0	+	+	0	0	+	+	0	0
Bu14	+	+	+	0	+	+	0	0	+	+	0	0	+	+	0	0	+	+	0	0
Bu15	+	+	+	+	+	+	+	+	+	+	+	0	+	+	+	+	+	+	+	0
Bu16	+	+	+	+	+	+	+	+	+	+	+	0	+	+	+		+	+	0	0
Bu17	+	+	0	0	+	+	0	0	+	+	0	0	+	+	0	0	+	+	0	0
Bu18	+	+	+	+	+	+	0	0	+	+	0	0	+	+	0	0	+	+	+	0
Bu19	+	+	+	+	+	+	0	0	+	+	+	0	+	+	0	0	+	+	0	0
Bu20	+	+	+	+	+	+	+	+	+	+	+	0	+	+	+	+	+	+	0	0
Bu21	+	+	+	+	+	+	0	0	+	+	0	0	+	+	0	0	+	+	+	0

Number	IFA-Titers																			
	TssM				OmpH				AhpC				BimA				Hcp1			
	1:8	1:16	1:32	1:64	1:8	1:16	1:32	1:64	1:8	1:16	1:32	1:64	1:8	1:16	1:32	1:64	1:8	1:16	1:32	1:64
Bu22	+	+	0	0	+	+	0	0	+	0	0	0	+	0	0	0	0	0	0	0
Bu23	+	+	+	+	+	+	0	0	+	+	0	0	+	+	0	0	+	+	0	0
Bu24	+	+	+	+	+	+	+	+	+	+	+	+	+	+	+	0	+	+	0	0
Bu25	+	+	+	+	+	+	+	+	+	+	+	0	+	+	0	0	+	+	0	0
Bu26	0	0	0	0	0	0	0	0	0	0	0	0	0	0	0	0	0	0	0	0
Bu27	+	+	+		+	+	0	0	+	+	0	0	+	+	0	0	+	+	0	0
Bu28	+	+	+	+	+	+	+	+	+	+	+	+	+	+	+	0	+	+	+	0
Bu29	+	+	+	+	+	+	+	0	+	+	0	0	+	+	0	0	+	+	0	0
Bu30	+	+	+	+	+	+	0	0	+	+	0	0	+	+	0	0	+	+	0	0
Bu31	+	+	+	+	+	+	+	+	+	+	+	+	+	+	+	+	+	+	+	+
Bu32	+	+	0	0	+	0	0	0	+	+	+	0	+	+	0	0	0	0	0	0
Bu33	+	+	0	0	+	+	0	0	+	+	0	0	+	+	0	0	0	0	0	0
Bu34	+	+	+	+	+	+	0	0	+	+	+	0	+	+	+	0	+	+	+	0
Bu35	+	+	+	0	+	+	0	0	+	+	0	0	+	0	0	0	+	+	0	0
Bu36	+	+	+	0	+	+	0	0	0	0	0	0	0	0	0	0	+	+	0	0
Bu37	+	+	+	+	+	+	+	+	+	+	+	0	+	+	0	0	+	+	0	0
Bu38	+	+	+	0	+	+	0	0	+	+	0	0	+	0	0	0	+	+	0	0
Bu39	+	+	0	0	+	+	0	0	0	0	0	0	0	0	0	0	0	0	0	0
Bu40	+	+	+	0	+	+	+	+	+	+	+	0	+	+	0	0	0	0	0	0
	38	38	32	21	37	37	18	8	36	36	21	4	36	33	12	4	32	32	10	2
**Mukdahan hospital**																				
Muk1	+	+	+	+	+	+	+	+	+	+	+	0	+	+	+	0	+	+	+	0
Muk2	+	+	+	+	+	+	+	+	+	+	0	0	+	+	+	0	+	+	0	0
Muk3	+	+	0	0	+	+	0	0	0	0	0	0	0	0	0	0	+	+	0	0

**Number**	**IFA-Titers**																			
	TssM				OmpH				AhpC				BimA				Hcp1			
	1:8	1:16	1:32	1:64	1:8	1:16	1:32	1:64	1:8	1:16	1:32	1:64	1:8	1:16	1:32	1:64	1:8	1:16	1:32	1:64
Muk4	+	+	+	+	+	+	0	0	+	+	0	0	+	+	0	0	+	+	0	0
Muk5	+	+	+	+	+	+	+	+	+	+	0	0	+	+	0	0	0	0	0	0
Muk6	+	+	+	+	+	+	+	+	+	+	+	+	+	+	+	+	+	+	+	+
Muk7	0	0	0	0	0	0	0	0	0	0	0	0	0	0	0	0	0	0	0	0
Muk8	+	+	+	+	+	+	+	+	+	+	+	0	+	+	+	0	+	+	+	0
Muk9	+	+	+	+	+	+	+	+	+	+	0	0	+	+	0	0	+	+	0	0
Muk10	+	+	+	+	+	+	+	+	+	+	0	0	+	+	0	0	+	+	0	0
Muk11	+	+	+	+	+	+	+	+	+	+	0	0	+	+	+	0	+	+	+	0
Muk12	+	+	+	+	+	+	0	0	+	+	0	0	0	0	0	0	0	0	0	0
Muk13	+	+	+	+	+	+	+	+	+	+	+	0	+	+	+	0	+	+	0	0
Muk14	+	+	0	0	+	+	0	0	+	+	0	0	+	+	0	0	0	0	0	0
Muk15	0	0	0	0	0	0	0	0	0	0	0	0	0	0	0	0	0	0	0	0
	13	13	11	5	13	13	9	4	12	12	4	1	11	11	6	1	10	10	4	1
**Chaiyaphum hospital**																				
Chai1	+	+	+	+	+	+	+	+	+	+	+	0	+	+	+	0	+	+	+	0
Chai2	+	+	+	+	+	+	0	0	0	0	0	0	0	0	0	0	0	0	0	0
Chai3	+	+	+	+	+	+	0	0	+	+	0	0	+	+	0	0	0	0	0	0
Chai4	+	+	+	+	+	+	+	+	+	+	+	0	+	+	+	0	+	+	+	0
Chai5	+	+	+	+	+	+	+	0	+	+	+	0	+	+	0	0	+	0	0	0
Chai6	+	+	0	0	0	0	0	0	0	0	0	0	0	0	0	0	0	0	0	0
Chai7	+	+	+	+	+	+	0	0	+	+	0	0	+	+	0	0	0	0	0	0
Chai8	+	+	+	+	+	+	+	0	+	+	+	0	+	+	+	0	0	0	0	0
Chai9	+	+	+	+	+	+	+	+	+	+	+	0	+	+	+	0	+	+	+	0
Chai10	+	+	+	+	+	+	0	0	+	+	0	0	+	+	0	0	0	0	0	0

Number	IFA-Titers																			
	TssM				OmpH				AhpC				BimA				Hcp1			
	1:8	1:16	1:32	1:64	1:8	1:16	1:32	1:64	1:8	1:16	1:32	1:64	1:8	1:16	1:32	1:64	1:8	1:16	1:32	1:64
Chai11	+	+	+	+	+	+	0	0	+	+	0	0	+	+	0	0	0	0	0	0
Chai12	+	+	+	+	+	+	+	0	+	+	+	0	+	+	0	0	0	0	0	0
Chai13	+	+	+	+	+	+	+	0	+	+	+	0	+	+	+	0	0	0	0	0
Chai14	+	+	+	+	+	+	+	+	+	+	+	+	+	+	+	+	+	+	+	0
Chai15	0	0	0	0	0	0	0	0	0	0	0	0	0	0	0	0	0	0	0	0
Chai16	+	+	+	+	+	+	+	0	+	+	+	0	+	+	+	0	0	0	0	0
Chai17	+	+	+	+	+	+	+	0	+	+	+	0	+	+	+	0	0	0	0	0
Chai18	+	+	+	+	+	+	0	0	+	+	0	0	+	+	0	0	0	0	0	0
Chai19	+	+	+	+	+	+	+	0	+	+	+	0	0	0	0	0	0	0	0	0
	18	18	17	11	17	17	11	4	16	16	11	1	15	15	8	1	5	4	4	0
**Trang hospital**																				
T1	+	+	+	0	+	+	+	0	+	+	0	0	+	+	0	0	+	+	0	0
T2	0	0	0	0	0	0	0	0	0	0	0	0	0	0	0	0	0	0	0	0
T3	+	+	+	0	+	+	+	0	+	+	+	0	+	+	+	0	+	+	+	0
T4	+	+	+	0	+	+	+	0	+	+	0	0	+	+	0	0	+	+	0	0
T5	+	+	+	0	+	+	0	0	+	+	0	0	+	+	0	0	0	0	0	0
T6	+	+	+	+	+	+	+	+	+	+	+	+	+	+	+	0	+	+	+	0
T7	+	+	0	0	0	0	0	0	+	0	0	0	0	0	0	0	0	0	0	0
	6	6	5	1	5	5	4	1	6	5	2	1	5	5	2	0	4	4	2	0
Total	75	75	65	38	72	72	42	17	70	69	38	7	67	64	28	6	51	50	20	3

Table S2. Result Anti-*B. pseudomallei* antibody detection from serums of other infection samples when testing by IFA that use TssM, OmpH, AhpC, BimA, and Hcp1 expressed *E. coli* as antigen

**Number**	**IFA-Titers**																			
	TssM				OmpH				AhpC				BimA				Hcp1			
	1:8	1:16	1:32	1:64	1:8	1:16	1:32	1:64	1:8	1:16	1:32	1:64	1:8	1:16	1:32	1:64	1:8	1:16	1:32	1:64
**Other infections:**																				
Pseudomonas spp.																				
1	0	0	0	0	0	0	0	0	+	0	0	0	0	0	0	0	+	0	0	0
2	0	0	0	0	0	0	0	0	0	0	0	0	0	0	0	0	0	0	0	0
3	0	0	0	0	0	0	0	0	0	0	0	0	0	0	0	0	0	0	0	0
4	0	0	0	0	0	0	0	0	0	0	0	0	0	0	0	0	0	0	0	0
Staphylococcus spp.																				
1	0	0	0	0	0	0	0	0	0	0	0	0	0	0	0	0	0	0	0	0
2	0	0	0	0	+	0	0	0	+	0	0	0	0	0	0	0	0	0	0	0
3	0	0	0	0	0	0	0	0	0	0	0	0	0	0	0	0	0	0	0	0
4	0	0	0	0	0	0	0	0	0	0	0	0	0	0	0	0	0	0	0	0
5	0	0	0	0	0	0	0	0	0	0	0	0	0	0	0	0	0	0	0	0
6	0	0	0	0	0	0	0	0	0	0	0	0	0	0	0	0	0	0	0	0
7	+	0	0	0	+	0	0	0	+	0	0	0	0	0	0	0	0	0	0	0
8	0	0	0	0	0	0	0	0	0	0	0	0	0	0	0	0	0	0	0	0
9	0	0	0	0	0	0	0	0	0	0	0	0	0	0	0	0	0	0	0	0
10	0	0	0	0	0	0	0	0	0	0	0	0	0	0	0	0	0	0	0	0
Streptococcus spp.																				
1	0	0	0	0	0	0	0	0	0	0	0	0	0	0	0	0	0	0	0	0
2	0	0	0	0	+	0	0	0	+	0	0	0	+	0	0	0	0	0	0	0
3	0	0	0	0	0	0	0	0	0	0	0	0	0	0	0	0	0	0	0	0
4	0	0	0	0	0	0	0	0	0	0	0	0	0	0	0	0	0	0	0	0

**Number**	**IFA-Titers**																			
	TssM				OmpH				AhpC				BimA				Hcp1			
	1:8	1:16	1:32	1:64	1:8	1:16	1:32	1:64	1:8	1:16	1:32	1:64	1:8	1:16	1:32	1:64	1:8	1:16	1:32	1:64
5	0	0	0	0	0	0	0	0	0	0	0	0	0	0	0	0	0	0	0	0
6	0	0	0	0	0	0	0	0	0	0	0	0	0	0	0	0	0	0	0	0
7	0	0	0	0	0	0	0	0	0	0	0	0	0	0	0	0	0	0	0	0
8	+	0	0	0	0	0	0	0	0	0	0	0	+	0	0	0	+	0	0	0
9	0	0	0	0	0	0	0	0	0	0	0	0	0	0	0	0	0	0	0	0
10	0	0	0	0	0	0	0	0	0	0	0	0	0	0	0	0	0	0	0	0
11	0	0	0	0	0	0	0	0	0	0	0	0	0	0	0	0	0	0	0	0
12	0	0	0	0	0	0	0	0	0	0	0	0	0	0	0	0	0	0	0	0
13	0	0	0	0	0	0	0	0	0	0	0	0	0	0	0	0	0	0	0	0
14	0	0	0	0	0	0	0	0	0	0	0	0	0	0	0	0	0	0	0	0
15	0	0	0	0	+	0	0	0	0	0	0	0	0	0	0	0	0	0	0	0
16	0	0	0	0	0	0	0	0	+	0	0	0	0	0	0	0	+	0	0	0
17	0	0	0	0	0	0	0	0	0	0	0	0	0	0	0	0	0	0	0	0
18	0	0	0	0	0	0	0	0	0	0	0	0	0	0	0	0	0	0	0	0
19	0	0	0	0	0	0	0	0	0	0	0	0	0	0	0	0	0	0	0	0
20	0	0	0	0	0	0	0	0	0	0	0	0	0	0	0	0	0	0	0	0
Escherichia coli																				
1	0	0	0	0	+	0	0	0	0	0	0	0	0	0	0	0	0	0	0	0
2	0	0	0	0	0	0	0	0	0	0	0	0	0	0	0	0	0	0	0	0
3	0	0	0	0	0	0	0	0	0	0	0	0	0	0	0	0	0	0	0	0
4	0	0	0	0	0	0	0	0	0	0	0	0	0	0	0	0	0	0	0	0
5	0	0	0	0	+	0	0	0	+	0	0	0	+	0	0	0	+	0	0	0
6	0	0	0	0	0	0	0	0	0	0	0	0	0	0	0	0	0	0	0	0
7	0	0	0	0	0	0	0	0	0	0	0	0	0	0	0	0	0	0	0	0

Number	IFA-Titers																			
	TssM				OmpH				AhpC				BimA				Hcp1			
	1:8	1:16	1:32	1:64	1:8	1:16	1:32	1:64	1:8	1:16	1:32	1:64	1:8	1:16	1:32	1:64	1:8	1:16	1:32	1:64
8	0	0	0	0	0	0	0	0	0	0	0	0	0	0	0	0	0	0	0	0
Bacillus spp.																				
1	0	0	0	0	0	0	0	0	0	0	0	0	+	0	0	0	0	0	0	0
2	0	0	0	0	+	0	0	0	0	0	0	0	0	0	0	0	0	0	0	0
3	0	0	0	0	0	0	0	0	0	0	0	0	0	0	0	0	0	0	0	0
4	0	0	0	0	0	0	0	0	0	0	0	0	0	0	0	0	0	0	0	0
5	0	0	0	0	0	0	0	0	0	0	0	0	0	0	0	0	0	0	0	0
6	0	0	0	0	0	0	0	0	0	0	0	0	0	0	0	0	0	0	0	0
Klebsiella spp.																				
1	0	0	0	0	0	0	0	0	+	0	0	0	0	0	0	0	0	0	0	0
2	0	0	0	0	0	0	0	0	0	0	0	0	0	0	0	0	0	0	0	0
Acinetobacter spp.																				
1	0	0	0	0	+	0	0	0	0	0	0	0	0	0	0	0	0	0	0	0
2	0	0	0	0	0	0	0	0	0	0	0	0	0	0	0	0	0	0	0	0
Proteus spp.																				
1	0	0	0	0	0	0	0	0	+	0	0	0	0	0	0	0	0	0	0	0
2	0	0	0	0	0	0	0	0	0	0	0	0	0	0	0	0	0	0	0	0
Salmonella spp.																				
1	0	0	0	0	0	0	0	0	0	0	0	0	+	0	0	0	0	0	0	0
2	0	0	0	0	0	0	0	0	0	0	0	0	0	0	0	0	0	0	0	0
HBV																				
1	0	0	0	0	0	0	0	0	0	0	0	0	0	0	0	0	0	0	0	0
2	0	0	0	0	0	0	0	0	+	0	0	0	0	0	0	0	0	0	0	0
3	0	0	0	0	0	0	0	0	0	0	0	0	0	0	0	0	0	0	0	0

Number	IFA-Titers																			
	TssM				OmpH				AhpC				BimA				Hcp1			
	1:8	1:16	1:32	1:64	1:8	1:16	1:32	1:64	1:8	1:16	1:32	1:64	1:8	1:16	1:32	1:64	1:8	1:16	1:32	1:64
HIV																				
1	0	0	0	0	0	0	0	0	0	0	0	0	0	0	0	0	0	0	0	0
2	0	0	0	0	0	0	0	0	0	0	0	0	0	0	0	0	0	0	0	0
3	0	0	0	0	+	0	0	0	0	0	0	0	+	0	0	0	0	0	0	0
4	0	0	0	0	0	0	0	0	0	0	0	0	0	0	0	0	0	0	0	0
5	0	0	0	0	0	0	0	0	0	0	0	0	0	0	0	0	0	0	0	0
6	0	0	0	0	0	0	0	0	0	0	0	0	0	0	0	0	0	0	0	0
7	0	0	0	0	0	0	0	0	0	0	0	0	0	0	0	0	0	0	0	0
8	0	0	0	0	+	0	0	0	0	0	0	0	+	0	0	0	0	0	0	0
9	0	0	0	0	0	0	0	0	0	0	0	0	0	0	0	0	0	0	0	0
10	0	0	0	0	0	0	0	0	0	0	0	0	0	0	0	0	0	0	0	0
11	0	0	0	0	0	0	0	0	0	0	0	0	0	0	0	0	0	0	0	0
Total	2	0	0	0	10	0	0	0	9	0	0	0	7	0	0	0	4	0	0	0

Table S3. Result Anti-*B. pseudomallei* antibody detection from serums of healthy donors samples when testing by IFA that use TssM, OmpH, AhpC, BimA, and Hcp1 expressed *E. coli* as antigen

**Number**	**IFA Titers**																			
	TssM				OmpH				AhpC				BimA				Hcp1			
	1:8	1:16	1:32	1:64	1:8	1:16	1:32	1:64	1:8	1:16	1:32	1:64	1:8	1:16	1:32	1:64	1:8	1:16	1:32	1:64
**Healthy donors**																				
1	0	0	0	0	0	0	0	0	0	0	0	0	0	0	0	0	0	0	0	0
2	0	0	0	0	0	0	0	0	0	0	0	0	0	0	0	0	0	0	0	0
3	0	0	0	0	0	0	0	0	0	0	0	0	0	0	0	0	0	0	0	0
4	0	0	0	0	0	0	0	0	0	0	0	0	0	0	0	0	0	0	0	0
5	0	0	0	0	0	0	0	0	0	0	0	0	0	0	0	0	0	0	0	0
6	0	0	0	0	0	0	0	0	0	0	0	0	0	0	0	0	0	0	0	0
7	0	0	0	0	0	0	0	0	0	0	0	0	0	0	0	0	0	0	0	0
8	0	0	0	0	0	0	0	0	0	0	0	0	0	0	0	0	0	0	0	0
9	0	0	0	0	0	0	0	0	0	0	0	0	0	0	0	0	0	0	0	0
10	0	0	0	0	0	0	0	0	0	0	0	0	0	0	0	0	0	0	0	0
11	0	0	0	0	0	0	0	0	0	0	0	0	0	0	0	0	0	0	0	0
12	0	0	0	0	0	0	0	0	0	0	0	0	0	0	0	0	0	0	0	0
13	0	0	0	0	0	0	0	0	0	0	0	0	0	0	0	0	0	0	0	0
14	0	0	0	0	0	0	0	0	0	0	0	0	0	0	0	0	0	0	0	0
15	0	0	0	0	0	0	0	0	0	0	0	0	0	0	0	0	0	0	0	0
16	0	0	0	0	0	0	0	0	0	0	0	0	0	0	0	0	0	0	0	0
17	0	0	0	0	0	0	0	0	0	0	0	0	0	0	0	0	0	0	0	0
18	0	0	0	0	0	0	0	0	0	0	0	0	0	0	0	0	0	0	0	0
19	0	0	0	0	0	0	0	0	0	0	0	0	0	0	0	0	0	0	0	0
20	0	0	0	0	0	0	0	0	0	0	0	0	0	0	0	0	0	0	0	0
21	0	0	0	0	0	0	0	0	0	0	0	0	0	0	0	0	0	0	0	0

Number	IFA Titers																			
	TssM				OmpH				AhpC				BimA				Hcp1			
	1:8	1:16	1:32	1:64	1:8	1:16	1:32	1:64	1:8	1:16	1:32	1:64	1:8	1:16	1:32	1:64	1:8	1:16	1:32	1:64
22	0	0	0	0	0	0	0	0	0	0	0	0	0	0	0	0	0	0	0	0
23	0	0	0	0	0	0	0	0	0	0	0	0	0	0	0	0	0	0	0	0
24	0	0	0	0	0	0	0	0	0	0	0	0	0	0	0	0	0	0	0	0
25	0	0	0	0	0	0	0	0	0	0	0	0	0	0	0	0	0	0	0	0
26	0	0	0	0	0	0	0	0	0	0	0	0	0	0	0	0	0	0	0	0
27	0	0	0	0	0	0	0	0	0	0	0	0	0	0	0	0	0	0	0	0
28	0	0	0	0	0	0	0	0	0	0	0	0	0	0	0	0	0	0	0	0
29	0	0	0	0	0	0	0	0	0	0	0	0	0	0	0	0	0	0	0	0
30	0	0	0	0	0	0	0	0	0	0	0	0	0	0	0	0	0	0	0	0
31	0	0	0	0	0	0	0	0	0	0	0	0	0	0	0	0	0	0	0	0
32	0	0	0	0	0	0	0	0	0	0	0	0	0	0	0	0	0	0	0	0
33	0	0	0	0	0	0	0	0	0	0	0	0	0	0	0	0	0	0	0	0
34	0	0	0	0	0	0	0	0	0	0	0	0	0	0	0	0	0	0	0	0
35	0	0	0	0	0	0	0	0	0	0	0	0	0	0	0	0	0	0	0	0
36	0	0	0	0	0	0	0	0	0	0	0	0	0	0	0	0	0	0	0	0
37	0	0	0	0	0	0	0	0	0	0	0	0	0	0	0	0	0	0	0	0
38	0	0	0	0	0	0	0	0	0	0	0	0	0	0	0	0	0	0	0	0
39	0	0	0	0	0	0	0	0	0	0	0	0	0	0	0	0	0	0	0	0
40	0	0	0	0	0	0	0	0	0	0	0	0	0	0	0	0	0	0	0	0
41	0	0	0	0	0	0	0	0	0	0	0	0	0	0	0	0	0	0	0	0
42	0	0	0	0	0	0	0	0	0	0	0	0	0	0	0	0	0	0	0	0
43	0	0	0	0	0	0	0	0	0	0	0	0	0	0	0	0	0	0	0	0
44	0	0	0	0	0	0	0	0	0	0	0	0	0	0	0	0	0	0	0	0
45	0	0	0	0	0	0	0	0	0	0	0	0	0	0	0	0	0	0	0	0

Number	IFA Titers																			
	TssM				OmpH				AhpC				BimA				Hcp1			
	1:8	1:16	1:32	1:64	1:8	1:16	1:32	1:64	1:8	1:16	1:32	1:64	1:8	1:16	1:32	1:64	1:8	1:16	1:32	1:64
46	0	0	0	0	0	0	0	0	0	0	0	0	0	0	0	0	0	0	0	0
47	0	0	0	0	0	0	0	0	0	0	0	0	0	0	0	0	0	0	0	0
48	0	0	0	0	0	0	0	0	0	0	0	0	0	0	0	0	0	0	0	0
49	0	0	0	0	0	0	0	0	0	0	0	0	0	0	0	0	0	0	0	0
50	0	0	0	0	0	0	0	0	0	0	0	0	0	0	0	0	0	0	0	0
51	0	0	0	0	0	0	0	0	0	0	0	0	0	0	0	0	0	0	0	0
52	0	0	0	0	0	0	0	0	0	0	0	0	0	0	0	0	0	0	0	0
53	0	0	0	0	0	0	0	0	0	0	0	0	0	0	0	0	0	0	0	0
54	0	0	0	0	0	0	0	0	0	0	0	0	0	0	0	0	0	0	0	0
55	0	0	0	0	0	0	0	0	0	0	0	0	0	0	0	0	0	0	0	0
56	0	0	0	0	0	0	0	0	0	0	0	0	0	0	0	0	0	0	0	0
57	0	0	0	0	0	0	0	0	0	0	0	0	0	0	0	0	0	0	0	0
58	0	0	0	0	0	0	0	0	0	0	0	0	0	0	0	0	0	0	0	0
59	0	0	0	0	0	0	0	0	0	0	0	0	0	0	0	0	0	0	0	0
60	0	0	0	0	0	0	0	0	0	0	0	0	0	0	0	0	0	0	0	0
61	0	0	0	0	0	0	0	0	0	0	0	0	0	0	0	0	0	0	0	0
62	0	0	0	0	0	0	0	0	0	0	0	0	0	0	0	0	0	0	0	0
63	0	0	0	0	0	0	0	0	1	0	0	0	0	0	0	0	0	0	0	0
64	0	0	0	0	0	0	0	0	0	0	0	0	0	0	0	0	0	0	0	0
65	0	0	0	0	0	0	0	0	0	0	0	0	0	0	0	0	0	0	0	0
66	0	0	0	0	0	0	0	0	0	0	0	0	0	0	0	0	0	0	0	0
67	0	0	0	0	0	0	0	0	0	0	0	0	0	0	0	0	0	0	0	0
68	0	0	0	0	0	0	0	0	0	0	0	0	0	0	0	0	0	0	0	0
79	0	0	0	0	0	0	0	0	0	0	0	0	0	0	0	0	0	0	0	0

Number	IFA Titers																			
	TssM				OmpH				AhpC				BimA				Hcp1			
	1:8	1:16	1:32	1:64	1:8	1:16	1:32	1:64	1:8	1:16	1:32	1:64	1:8	1:16	1:32	1:64	1:8	1:16	1:32	1:64
70	0	0	0	0	0	0	0	0	0	0	0	0	0	0	0	0	0	0	0	0
71	0	0	0	0	0	0	0	0	0	0	0	0	0	0	0	0	0	0	0	0
72	0	0	0	0	0	0	0	0	0	0	0	0	0	0	0	0	0	0	0	0
73	0	0	0	0	0	0	0	0	0	0	0	0	0	0	0	0	0	0	0	0
74	0	0	0	0	0	0	0	0	0	0	0	0	0	0	0	0	0	0	0	0
75	0	0	0	0	0	0	0	0	0	0	0	0	0	0	0	0	0	0	0	0
76	0	0	0	0	0	0	0	0	0	0	0	0	0	0	0	0	0	0	0	0
77	0	0	0	0	0	0	0	0	0	0	0	0	0	0	0	0	0	0	0	0
78	0	0	0	0	0	0	0	0	0	0	0	0	0	0	0	0	0	0	0	0
79	0	0	0	0	0	0	0	0	0	0	0	0	0	0	0	0	0	0	0	0
80	0	0	0	0	0	0	0	0	0	0	0	0	0	0	0	0	0	0	0	0
81	0	0	0	0	0	0	0	0	0	0	0	0	0	0	0	0	0	0	0	0
82	0	0	0	0	0	0	0	0	0	0	0	0	0	0	0	0	0	0	0	0
83	0	0	0	0	0	0	0	0	0	0	0	0	0	0	0	0	0	0	0	0
84	0	0	0	0	0	0	0	0	0	0	0	0	0	0	0	0	0	0	0	0
85	0	0	0	0	0	0	0	0	0	0	0	0	0	0	0	0	0	0	0	0
86	0	0	0	0	0	0	0	0	0	0	0	0	0	0	0	0	0	0	0	0
87	0	0	0	0	0	0	0	0	0	0	0	0	0	0	0	0	0	0	0	0
98	0	0	0	0	0	0	0	0	0	0	0	0	0	0	0	0	0	0	0	0
99	0	0	0	0	0	0	0	0	0	0	0	0	0	0	0	0	0	0	0	0
90	0	0	0	0	0	0	0	0	0	0	0	0	0	0	0	0	0	0	0	0
91	0	0	0	0	0	0	0	0	0	0	0	0	0	0	0	0	0	0	0	0
92	0	0	0	0	0	0	0	0	0	0	0	0	0	0	0	0	0	0	0	0
93	0	0	0	0	0	0	0	0	0	0	0	0	0	0	0	0	0	0	0	0

Number	IFA Titers																			
	TssM				OmpH				AhpC				BimA				Hcp1			
	1:8	1:16	1:32	1:64	1:8	1:16	1:32	1:64	1:8	1:16	1:32	1:64	1:8	1:16	1:32	1:64	1:8	1:16	1:32	1:64
94	0	0	0	0	0	0	0	0	0	0	0	0	0	0	0	0	0	0	0	0
95	0	0	0	0	0	0	0	0	0	0	0	0	0	0	0	0	0	0	0	0
96	0	0	0	0	0	0	0	0	0	0	0	0	0	0	0	0	0	0	0	0
100	0	0	0	0	0	0	0	0	0	0	0	0	0	0	0	0	0	0	0	0
101	0	0	0	0	0	0	0	0	0	0	0	0	0	0	0	0	0	0	0	0
102	0	0	0	0	0	0	0	0	0	0	0	0	0	0	0	0	0	0	0	0
103	0	0	0	0	0	0	0	0	0	0	0	0	0	0	0	0	0	0	0	0
104	0	0	0	0	0	0	0	0	0	0	0	0	0	0	0	0	0	0	0	0
105	0	0	0	0	0	0	0	0	0	0	0	0	0	0	0	0	0	0	0	0
106	0	0	0	0	0	0	0	0	0	0	0	0	0	0	0	0	0	0	0	0
107	0	0	0	0	0	0	0	0	0	0	0	0	0	0	0	0	0	0	0	0
108	0	0	0	0	0	0	0	0	0	0	0	0	0	0	0	0	0	0	0	0
109	0	0	0	0	0	0	0	0	0	0	0	0	0	0	0	0	0	0	0	0
110	0	0	0	0	0	0	0	0	0	0	0	0	0	0	0	0	0	0	0	0
111	0	0	0	0	0	0	0	0	0	0	0	0	0	0	0	0	0	0	0	0
112	0	0	0	0	0	0	0	0	0	0	0	0	0	0	0	0	0	0	0	0
113	0	0	0	0	0	0	0	0	0	0	0	0	0	0	0	0	0	0	0	0
114	0	0	0	0	0	0	0	0	0	0	0	0	0	0	0	0	0	0	0	0
115	0	0	0	0	0	0	0	0	0	0	0	0	0	0	0	0	0	0	0	0
116	0	0	0	0	0	0	0	0	0	0	0	0	0	0	0	0	0	0	0	0
117	0	0	0	0	0	0	0	0	0	0	0	0	0	0	0	0	0	0	0	0
118	0	0	0	0	0	0	0	0	0	0	0	0	0	0	0	0	0	0	0	0
119	0	0	0	0	0	0	0	0	0	0	0	0	0	0	0	0	0	0	0	0
120	0	0	0	0	0	0	0	0	0	0	0	0	0	0	0	0	0	0	0	0

**Number**	**IFA Titers**																			
	TssM				OmpH				AhpC				BimA				Hcp1			
	1:8	1:16	1:32	1:64	1:8	1:16	1:32	1:64	1:8	1:16	1:32	1:64	1:8	1:16	1:32	1:64	1:8	1:16	1:32	1:64
Total	0	0	0	0	0	0	0	0	1	0	0	0	0	0	0	0	0	0	0	0
